# The role of epithelial membrane antigen (EMA) overexpression in the prognosis of prostatic adenocarcinoma

**DOI:** 10.25122/jml-2021-0272

**Published:** 2022-04

**Authors:** Rawaa Ghalib, Athraa Falah

**Affiliations:** 1. Department of Pathology, College of Medicine, Babylon University, Babylon, Iraq

**Keywords:** epithelial membrane antigen, overexpression, prognosis, prostatic adenocarcinoma

## Abstract

Prostatic adenocarcinoma is the second-most common cancer in men and the fifth most common cause of cancer death. Its incidence increases with age; 75% of patients are 65 years and older. The aim of the study was to assess epithelial membrane antigen (EMA) expression in prostatic adenocarcinoma as a poor prognostic marker and its correlation to some pathological parameters. The formalin-fixed, paraffin-surrounded tissue blocks were retrospectively collected from 40 men diagnosed with prostate carcinoma. All cases were collected from Al Hilla Teaching Hospital and some private labs between October 2018 – November 2020, with ages ranging from 30–89 years. Statistical analysis was done using SPSS 22, frequency and percentage were used for categorical data, and Chi-square was used to evaluate connotation between variables. P-value ≤0.05 was significant. The blocks were sectioned for EMA immunohistochemical staining using monoclonal mouse anti-human EMA protein. EMA protein overexpression was detected in 75% (n=30/40) of prostatic adenocarcinoma cases. EMA expression showed no correlation with the patient's age (P=0.09) and a positive correlation with the cancer grade (P=0.003). In prostatic adenocarcinoma patients, EMA could be seen as a potential prognostic predictor for disease progression.

## INTRODUCTION

Prostatic adenocarcinoma is the second-most common cancer in men and the fifth most common cause of cancer death [1]. Its incidence increases with age; 75% of patients are 65 years and older [2]. Environmental and hereditary features play a part in the pathogenesis; males associated with 1^st^-degree families have 10 times higher risk [3]. Increased androgen levels accelerate prostate cancer progress, and most prostatic adenocarcinomas are asymptomatic during PSA screening [4]. There is a low 5-year survival rate in patients with prostatic adenocarcinoma (34%); therefore, we need new methods for early detection and target therapy [5, 6]. Epithelial membrane antigen (EMA), also called polymorphic epithelial mucin (PEM), is a member of a family of transmembrane mucin glycoproteins found on chromosome 1q22 (MUC1 gene). It consists of a constant cytoplasmic domain of 69 amino acids and an extracellular domain of 20 amino acid tandem repeats of serine and threonine residues with several O-linked oligosaccharide side chains with high molecular weights of 250–500 kDa [7]. EMA epitope is situated on several tissues of the apical cell surface of the normal glandular epithelium. The protein supports the cell in defense against pathogens, plays an essential role in a cell signaling capacity, and inhibits the production of E-cadherin/beta-catenin, which are derived from the mammary epithelium [8]. Changes in MUC1-EMA expression, secretion, and glycosylation are typically related to colon, breast, ovarian, lung, and pancreatic cancer as a poor prognostic factor. Therefore these malignancy-associated recommend that MUC1 epitopes may be used as a target for anticancer therapies [9, 10]. The aim of the study was to assess epithelial membrane antigen (EMA) expression in prostatic adenocarcinoma as a poor prognostic marker and its correlation to some pathological parameters.

## Material and Methods

### Samples

In this retrospective cross-sectional study, we collected formalin-fixed, paraffin-embedded tissue blocks. Histologically, prostatic adenocarcinoma samples were collected randomly from the histopathology laboratory in Al-Hilla Teaching Hospital and private laboratories in this province from October 2018- to November 2020.

### Clinical and pathological data of the patients

The clinicopathological assessment of patients' ages ranges from 30 to 89 years. Patients were categorized as follows: five cases in age group 30–49y, fifteen cases in age group 50–69y, and twenty cases in age group 70–89y. According to the Gleason grading system, the cases are subdivided into 8, 15, 17 in relation to 6, 7, 7–10 grades [11]. 

### Immunohistochemistry of EMA in prostatic adenocarcinoma tissues

The automated immunohistochemical staining system was applied to determine the expression of EMA protein on the formalin-fixed paraffin-embedded blocks using primary antibody EMA (Monoclonal Mouse Anti-Human EMA protein, Dako Denmark A/S) (1:100) and the staining kit (DakoCytomation). The apical cytoplasmic staining results were recorded as staining scores, from 0–3, as follows: 

0: staining in <5% of tumor cells;

1: weak staining in ≥5%;

2: moderate staining in ≥5%;

3: strong staining in ≥5% of the tumor cells.

Positive staining was defined as a staining score of 2 or 3, while negative staining was defined as a score of 0 and 1 [11]. Statistical analysis was done using SPSS 22, frequency and percentage were used for categorical data, and Chi-square was used to evaluate connotation between variables. P-value ≤0.05 is significant.

## Results

### Association between EMA protein overexpression and aging group of patients with prostatic adenocarcinoma

**Figure 1.**
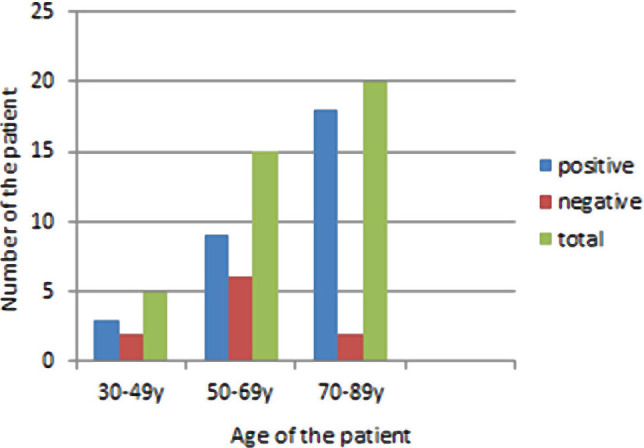
Distribution of EMA in prostatic adenocarcinoma in relation to the age of the patient.

EMA protein overexpression was noted in 75% (30 cases out of 40 cases) of prostatic adenocarcinoma (Table 1). Approximately 60% (n=3) of patients in age group 30–49y with prostatic adenocarcinoma had positive overexpression of EMA protein, while 40% (n=2) exhibited negative expression. Similarly, 60% (n=9) of patients in age group 50–69y with prostatic adenocarcinoma showed positive overexpression of EMA protein, whereas 40% (n=6) exhibited negative expression and 90% (n=18) of patients in age group 70–89y with prostatic adenocarcinoma showed positive overexpression of EMA protein whereas 10% (n=2) exhibited negative expression. There was an increase in the rate of EMA protein overexpression in relation to the increase in the age group, but there was no positive correlation between EMA protein overexpression and the aging group (P=0.09) (Figure 1).

**Table 1.** EMA immunostaining in relation to age of the patient, grade of the tumor in prostatic adenocarcinoma.

**Parameter**	**Immunostaining of EMA**		**Total**	**P-value**
	**Positive**	**Negative**		
**Age of the patient**				
30–49y	3 (60%)	2 (40%)	5 (12.5%)	P=0.09
50–69y	9 (60%)	6 (40%)	15 (37.5%)	
70–89y	18 (90%)	2 (10%)	20 (50%)	
**Gleason grading**				
6	5 (62.5%)	3 (37.5%)	8 (20%)	P=0.003
7	10 (66.7%)	5 (33.3%)	15 (37.5%)	
7–10	15 (88.2%)	2(11.8)	17 (42.5%)	
Total	30 (75%)	10 (25%)	40 (100%)	

P-value≤0.05 (significant).

### Association between EMA protein overexpression and clinical grading of prostatic adenocarcinoma

**Figure 2.**
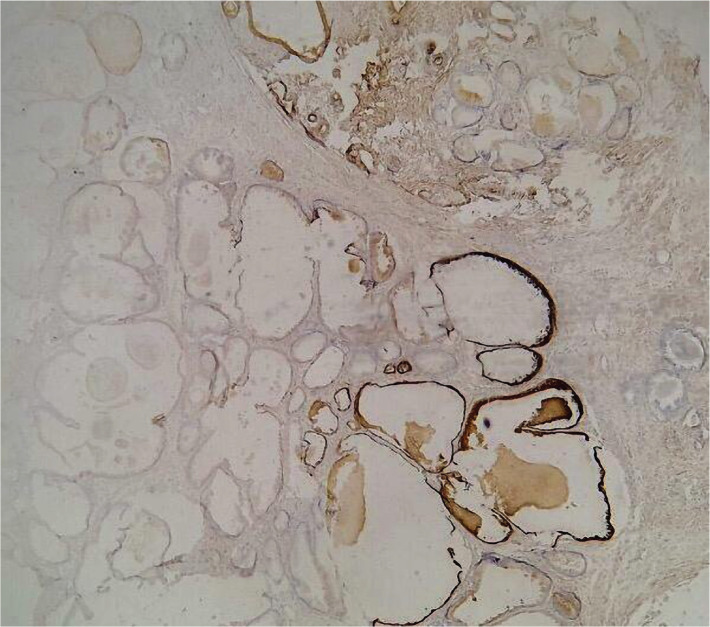
Prostatic adenocarcinoma showing moderate staining in ≥5% of pattern EMA (10X).

**Figure 3.**
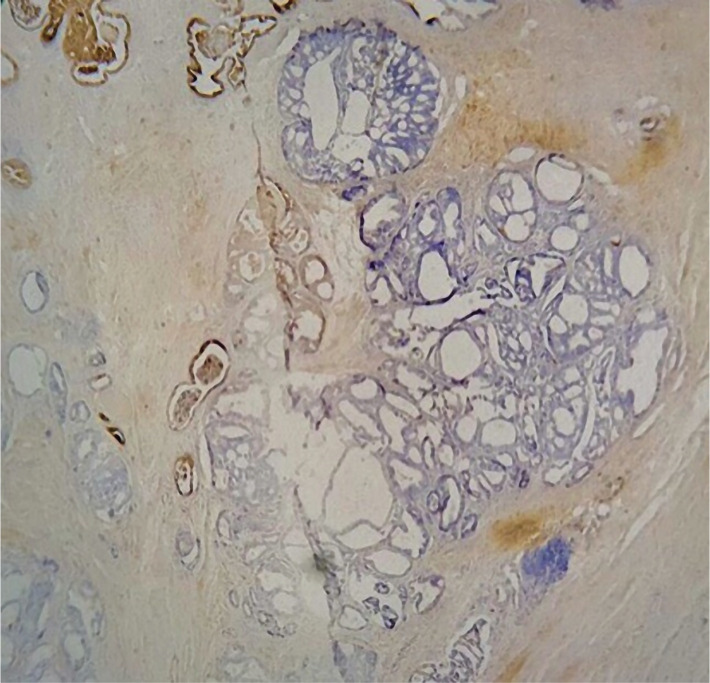
Prostatic adenocarcinoma IHC showing negative staining pattern of EMA (10X).

**Figure 4.**
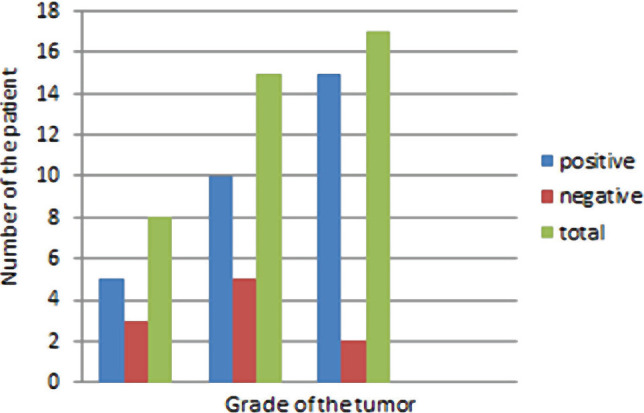
Distribution of EMA in prostatic adenocarcinoma in relation to the grade of the tumor.

Table 1 shows the correlation between the overexpression of EMA protein and the grade of the tumor. According to the Gleason score, 62.5% (n=5) of patients with prostatic adenocarcinoma in grade 6 showed positive overexpression of EMA protein, whereas 37.5% (n=3) exhibited negative expression. 66.7% (n=10) of patients with prostatic adenocarcinoma in grade 7 showed positive overexpression of EMA protein (Figure 2), whereas 33.3% (n=5) exhibited negative expression (Figure 3). Furthermore, 88.2% (n=15) of patients with prostatic adenocarcinoma in grades 7–10 showed positive overexpression of EMA protein, whereas 11.8% (n=2) exhibited negative expression, so there was a significant positive correlation between EMA protein expressions and the grade of the tumor (p=0.003) (Figure 4).

## Discussion

MUC1 glycoprotein is presented with different types used for diagnosis, staging, and therapy in certain forms of epithelial cancers. Prostatic adenocarcinoma is very common in Iraq, it has been widely distributed among the middle and old age group since the normal and abnormal prostatic tissue expresses different MUC1 epitopes, but MUC1 epitopes specific to prostate cancer have not been well characterized. In our study, we use a single anti-MUC1 epitope, EMA, to understand its role in the prognosis of the disease and its correlation with Gleason grades and the age of the patients [12]. Most forms of carcinomas overexpress MUC1 with various epitopes being spread over the cell surface. These abnormal glycans result from incomplete glycosylation and premature sialylation. There are different expression levels of MUC1 –EMA epitopes that can be recognized in prostatic adenocarcinoma tissue compared with healthy cells [13, 14].

The detection rate of EMA overexpression is 75% (30 out of 40), and our findings show a significant positive correlation with EMA overexpression as increasing with the grade according to the Gleason score. There are 62.5% (n=5) of patients with prostatic adenocarcinoma in grade 6 showing positive overexpression of EMA protein, 66.7% (n=10) with prostatic adenocarcinoma in grade 7 showing positive overexpression of EMA protein and 88.2% (n=15) with prostatic adenocarcinoma in grade 7–10 showing positive overexpression of EMA protein. The results of this study state a positive correlation between EMA protein expressions and the grade of the tumor (p=0.003). Similar to other studies, these results found that EMA overexpression in high-grade prostatic adenocarcinoma is correlated with a poor prognosis and might play a role in the progression and metastasis of the disease [15, 16]. The results of this study explain MUC1-EMA epitope may be associated with a poor prognosis that might be a strong relationship with tumor angiogenesis required not only for continued tumor growth but also for invasion and metastasis and may be modified by androgens and estrogens receptors [17, 18].

Moreover, our result agrees with another study that found that higher Gleason grades were associated with markedly increased cellular staining (EMA, p=0.009). These results support targeting hypo glycosylated MUC1 epitopes in prostate cancer for more specific imaging and therapy applications [12]. The increased intracellular localization of EMA protein and the changes in glycosylation of this protein were related to carcinomas in poorly differentiated cases [19, 20]. This could explain the resistance of the tumor to the chemotherapeutic drugs, which are withdrawn by the strong glycosylation in the MUC1-EMA epitope extracellular domain. EMA overexpression is usually associated with high-grade prostatic adenocarcinoma, aggressiveness, and poor prognosis. These cases show resistance to the chemotherapeutic drugs by changing the glycosylation yields a heavy hydrophilic region that stimulates cancer cell growth. [21]. Also, EMA overexpression can interfere with or suppress the immunological activity of immune cells that facilitate tumor cells which may either secrete proteolytic enzymes themselves or induce stromal cells to elaborate proteases for invasion and metastasis [22].

## Conclusion

In prostatic adenocarcinoma patients, our study effectively added details that hyperglycosylated MUC1-EMA epitopes play a significant role in the progression of the disease and their correlation with high-grade Gleason grades. Thus, further studies on the EMA and other MUC1 epitopes are needed to attain additional diagnostic and prognostic information.

## Acknowledgments

### Conflict of interest

The authors declare no conflict of interest.

### Ethical approval

The study was approved by the ethical committee of the Faculty of Medicine, University of Babylon (103- 9^th^ March 2018).

### Data availability

Further data is available from the corresponding author on reasonable request.

### Authorship

RG contributed to data collection. AF contributed to writing the original draft, methodology, and editing. RG contributed to conceptualizing, and AF contributed to data analysis.
